# Baseline Assessment of Health Research Systems in Saudi Arabia: Harnessing Efforts and Mobilizing Actions

**DOI:** 10.1007/s44197-022-00058-0

**Published:** 2022-09-27

**Authors:** Abdullah A. Alfawaz, Khalid A. Salman, Fatimah H. Alotaibi, Faisal S. Almogbel, Dania Al-Jaroudi, Maily J. Alrowily, Abdulrahman B. Derkaoui, Abdulrahman S. Alqahtani, Racha Fadlallah, Diana Jamal, Fadi El-Jardali, Ziad A. Memish

**Affiliations:** 1 Health Research Program, Model of Care, Center of Excellence, Health Holding Company, Ministry of Health, Riyadh, Kingdom of Saudi Arabia; 2 Executive Director of Research and Knowledge Economy, Makkah Healthcare Cluster, Executive Director of Research and Innovation, King Abdullah Medical City in the Holy Capital, Ministry of Health, Makkah, Kingdom of Saudi Arabia; 3 Executive Director of Academic and Training Affairs, Director Research Department, AlHasa Health Cluster, Ministry of Health, Al Hofuf, Kingdom of Saudi Arabia; 4 Director Research Department, King Fahad Specialist Hospital, Ministry of Health, Buraidah, Kingdom of Saudi Arabia; 5 Reproductive Endocrinology and Infertility Consultant, Minimally Invasive Gynecologic Surgery, Reproductive Endocrinology and Infertility Medicine Department, Obstetrics and Gynecology, King Fahad Medical City, Riyadh, Kingdom of Saudi Arabia; 6 Executive Director of Research Center, Research Center, King Fahad Medical City, Ministry of Health, Riyadh, Kingdom of Saudi Arabia; 7 Director of the Research Center and Studies, Health Research Program (HRP) Cluster Leader (Aljouf), Ministry of Health, Sakakah, Kingdom of Saudi Arabia; 8 Population Health Management Director, Hail Health Cluster, Ministry of Health, Hail Health Cluster, Kingdom of Saudi Arabia; 9 VP of Transformation at the Health Holding Company, Ministry of Health, Riyadh, Kingdom of Saudi Arabia; 10 Faculty of Health Sciences, Department of Health Management and Policy, American University of Beirut, Beirut, Lebanon; 11 Department of Health Management and Policy. Faculty of Health Sciences, American University of Beirut, Beirut, Lebanon; 12 Knowledge to Policy Center, Faculty of Health Sciences, American University of Beirut, Beirut, Lebanon; 13 Department of Health Research Methods, Evidence, and Impact, McMaster University, Hamilton, ON Canada; 14 Research and Innovation Centre, King Saud Medical City, Ministry of Health and College of Medicine, AlFaisal University, Riyadh, Kingdom of Saudi Arabia; 15 Hubert Department of Global Health, Rollins School of Public Health, Emory University, Atlanta, GA USA

**Keywords:** Health System Research, Policy, Vision 2030, Ministry of Health, Saudi Arabia, Research governance, Health transformation, Health vision, Health sector, Kingdom of Saudi Arabia

## Abstract

Major transformations are taking place in the Kingdom of Saudi Arabia (KSA) to achieve the 2030 vision for the health sector. A key component in strengthening the health system is a strong research governance strategy that can support the decision-making process by providing timely and accurate evidence that reflects local context and needs. This paper sought to better understand governance structures and policies for health research systems and support clusters so that they function effectively. This paper outlines the findings of an in-depth baseline assessment of existing health research efforts, activities, and plans of eight research clusters in the KSA and identifies key gaps and strengths in health research governance and capabilities. A cross-sectional design was used to survey research clusters in KSA. A six-part survey was developed to better understand the research clusters’ health research governance and capacities. The survey was sent to all KSA clusters and was completed in a group setting during meetings. Findings clearly show strong efforts to support research governance initiatives in health clusters in KSA. While some clusters are more advanced than others, there are plenty of opportunities to share knowledge and combine efforts to help achieve the goals set out for KSA health transformation. This baseline assessment also reflects the first attempt of its kind to understand the KSA experience and provide much-needed lessons on country-wide efforts to support the health system given the trickling effect of this sector on all others, enhancing and advancing national growth.

## Background

Research governance comes from the process adopted by governments or institutions to ensure that activities are based on predetermined protocols to achieve accountability. Research must be governed at all stages, and research governance entails the implementation of the principles, standards, and requirements of a study, including the promotion of good research culture and practice [1–3]. England, Scotland, and Australia are some of the countries that have adopted a research governance act, others may also apply research governance principles, strategies, and frameworks to future projects (National Health and Medical Research Council (NHMRC), [14].

In the UK, the National Institute for Health and Care Excellence (NICE) implemented its national research governance policy in 2018 [8]. The policy was reviewed and updated in late 2021 (Jonsson & Bouvy [9]). The policy was implemented to create good practices and ensure high-quality research while applying all the necessary regulations. It highlights the importance of identifying the roles and responsibilities of all staff involved in a research project (including data collection, site-specific staff members, research, participants, etc.) [11]. It also illustrates the research governance framework and describes its implementation process [5, 6]. It includes the mandatory registration of all research activities, compulsory informed consent for studies involving human participants; information transparency for all research team members and participants; a detailed data procedure especially for projects that use third-party data, including the reporting of research results and research misconduct; and ethical review and research governance considerations to protect research participants [9].

In the Kingdom of Saudi Arabia (KSA), major transformations are taking place in efforts to achieve the 2030 vision for the health sector, which requires urgent action and new initiatives to improve healthcare services focusing on reforms to ease health service, enhance the quality and efficiency of health-care services, and strengthen the prevention of health threats. Realizing such ambitious goals requires effective leadership to navigate the health sector and steer transformation at both the organization and system levels. A key component in strengthening the health system is a strong research governance strategy that can support the decision-making process by providing timely and accurate evidence that reflects local context and needs.

As part of the KSA’s national health strategy, one solution to facilitate and integrate health system solutions was to divide the country into health clusters, which was conducted in waves (starting 2018) to support the gradual implementation of health system reforms. Because these different clusters were established at different times, they have varying levels of capacity and preparedness for conducting health systems research. They also address the needs of different population groups, and as such, their research agenda must reflect context-specific needs (Ministry of Health, [12].

Thus, to support the implementation of a research governance strategy, it is essential that the needs, existing resources, and capacities be assessed to identify overarching areas that must be addressed. Different clusters work on research agendas based on their capacities and contextual needs. Identifying common areas for collaboration can support a country-wide research agenda. This assessment can also help identify cluster-specific capacity-building needs and improvement areas.

## Objective

This paper outlines the findings of an in-depth baseline assessment of existing health research efforts, activities, and plans of eight research clusters in the KSA and identifies key gaps and strengths in health research governance and capabilities.

Specifically, this paper sought to better understand governance structures and policies for health research systems and support clusters so that they function effectively. Based on the findings, the clusters would help identify overarching policies and structures that can be applied to all of them as well as specific areas for individual ones. Another outcome would be a unified research agenda for clusters and potential areas for collaboration on research initiatives.

## Methodology

This study adopted a cross-sectional design that surveyed research clusters in KSA to address the above study objectives. The following summarizes the survey tool, sampling, and data analysis [15, 16].

### Tool and Sampling

A six-part survey was developed to better understand the research clusters’ health research governance and capacities. The tool was adapted from a framework developed by Pang et al. [17] for building the foundations of health research systems. Based on current literature and local contextual needs, a comprehensive survey was developed to include:

- Cluster characteristics. These include region, establishment year, size, and research type.
- Stewardship and governance. This section pertains to the availability of policies, guidelines, and procedures for health research, including its management process, in addition to issues on priority setting, ethical review structures, and monitoring and evaluation (M&E).
- Health research funding. This includes funding sources for ongoing research including spending and processes for calls for proposals and the capacity to manage funding.
- Resources, training, and capacity building. This section includes questions on technical and human resources, and training and capacity building.
- Health research production and use. Questions in this section include the number of projects conducted, number of submitted proposals, number and types of publications, capacity for knowledge translation, and means of dissemination.
- Open-ended question. This section focuses on respondents’ opinions on barriers to and facilitators of health research stewardship and governance, funding health research resources (human and physical), and health research production and use.

The survey was sent to all KSA clusters and was completed in a group setting during meetings coordinated by cluster leads between June and August 2021.

### Ethical Clearance

The study was exempted from ethical review, as it gathered information regarding cluster performance and did not collect personal information or identifiers of individuals working in the clusters or receiving health-care services there [18].

### Data Management and Analysis

Data was encoded and analyzed using Microsoft Excel. To maximize the utility of the findings, we employed different data visualization methods to outline the survey results. Univariate analysis was used to report the findings, outlined in tables and charts.

The qualitative component included thematic analysis, which summarized the collated responses. The team then examined the data more thoroughly to derive the main themes under barriers to and facilitators of the strengthening of health research systems within and across the clusters under each section outlined above.

## Results

Surveys were collected from eight research clusters in KSA, and the results for each section are outlined below.

### Cluster Characteristics

The clusters were recently established, with the first dating back to 2015. The most prominent types of research undertaken by these clusters were clinical and health service research (100% for both), social research (87.5%), and basic research (75%). The most commonly reported topics researched by clusters included coronavirus disease 2019 (COVID-19), cancer (mostly breast cancer in addition to cervical and ovarian cancer), respiratory diseases (including asthma, allergies, and Middle East Respiratory Syndrome coronavirus (MERS-Cov)), and patient safety issues (including quality and safety initiatives, medication and medical errors).

### Stewardship and Governance

This section discusses the results below, divided into five parts.

### Policies, Guidelines, or Procedures for Health Research

As per Table 1 below, not all health research policies and guidelines were available across clusters; when they are, they were mostly institution-specific. Notably, 50% of the clusters reported having policies for the governance of health research, data protection, and collaborative research but only at the institutional level and not the cluster level. Several clusters do not have certain policies in place, such as a code for good research practice and scientific integrity (50%); quality assurance (50%); risk management (50%); biospecimen access, use, and retention (75%); clinical trials (50%); authorship (50%); research misconduct (50%); sponsorship policies (50%); and research dissemination and knowledge translation (50%) Table 2.

**Table 1:** Health research policies, guidelines, and procedures

	Cluster level *N* (%)	Institution-specific *N* (%)	Institution-specific *N* (%)
*Indicate whether the below policies exist at the cluster or institutional levels*			
Governance of health research	1 (12.5%)	4 (50%)	3 (37.5%)
Code for good research practice and scientific integrity	1 (12.5%)	3 (37.5%)	4 (50%)
Data protection policy	2 (25%)	4 (50%)	2 (25%)
Data management, storage, and privacy	2 (25%)	3 (37.5%)	3 (37.5%)
Quality assurance and quality improvement	1 (12.5%)	3 (37.5%)	4 (50%)
Bio-specimen access, use, and retention	0 (0%)	2 (25%)	6 (75%)
Risk management, privacy, and safety	2 (25%)	2 (25%)	4 (50%)
Clinical trials–agreements, insurance, and indemnity	1 (12.5%)	3 (37.5%)	4 (50%)
Collaborative research	2 (25%)	4 (50%)	2 (25%)
Authorship, acknowledgment, and affiliation	1 (12.5%)	3 (37.5%)	4 (50%)
Confidentiality and intellectual property (IP)	2 (25%)	3 (37.5%)	3 (37.5%)
Research reporting (oversight)	2 (25%)	3 (37.5%)	3 (37.5%)
Research misconduct, disputes, and complaints management	1 (12.5%)	3 (37.5%)	4 (50%)
Sponsorship policy for health research	1 (12.5%)	3 (37.5%)	4 (50%)
Guidelines for the financial management of research funds	2 (25%)	3 (37.5%)	3 (37.5%)
Research dissemination and knowledge translation guidelines	2 (25%)	2 (25%)	4 (50%)

**Table 2:** Research ethics policies

	Cluster level *N* (%)	Institution-specific *N* (%)	Cluster- and institution-specific (%)	Not available (%)
*Research ethics policies*				
Establishment of ethics review committee	3 (37.5%)	4 (50%)	1 (12.5%)	0 (0%)
Membership composition and functions of ethics review committee	3 (37.5%)	4 (50%)	1 (12.5%)	0 (0%)
Protocol submission to ethics review committee	3 (37.5%)	4 (50%)	1 (12.5%)	0 (0%)
Review of a new research proposal and continuing review	3 (37.5%)	4 (50%)	1 (12.5%)	0 (0%)
Consent process and subject informed	3 (37.5%)	4 (50%)	1 (12.5%)	0 (0%)
Rights and protection of study participants	3 (37.5%)	4 (50%)	1 (12.5%)	0 (0%)
Management of conflict of interest	2 (25%)	4 (50%)	1 (12.5%)	1 (12.5%)
Research team role definitions	2 (25%)	4 (50%)	1 (12.5%)	1 (12.5%)

Similarly, regarding research ethics, 50% of the clusters reported establishing several of these policies at the institutional level. Only one cluster had all policies at both cluster and institutional levels.

### Research Governance and Management

Table 3 shows that 62.5% of the clusters have defined roles and responsibilities for health research and policies governing the health research conduct of their institutions. Meanwhile, 50% have processes for coordinating health research activities and projects across institutions, and 37.5% had an organogram for key entities and actors involved in health research governance. Two clusters (25%) reported adopting coordination processes for health research activities and projects between country clusters.

**Table 3:** Research governance and management

	*N* (%)
Organogram for key entities and actors involved in health research governance	3 (37.5%)
Health research defined roles and responsibilities	5 (62.5%)
Policies governing health research conduct within the institutions	5 (62.5%)
Process coordinating health research activities and projects across institutions	4 (50%)
Process coordinating health research activities and projects between country clusters	2 (25%)

### Priority Setting for Health Research

As Table 4 presents, only one cluster (12.5%) has a designated entity that identified research priorities, and two (25%) have bodies that fund health research. Two clusters (25%) use institution-specific tools and approaches to identify research priorities. In addition, further two clusters (25%) have processes in place to assess population needs within the cluster and updated lists of national health research priorities for institutions. Only 25% of the clusters acknowledged that their projects address local priorities, while 62.5% agreed and strongly agreed that their projects concentrate on national priorities Table 5.

**Table 4:** Priority setting for health research

	Yes *N* (%)	No *N* (%)
Does the cluster have a designated entity that identifies health research priorities?	1 (12.5%)	7 (87.5%)
Is there a standardized process for assessing population needs within the cluster?	2 (25%)	6 (75%)
Is there a mechanism to communicate local health research priorities with different institutions in your cluster?	1 (12.5%)	7 (87.5%)
Is there a mechanism to communicate local health research priorities with other clusters?	1 (12.5%)	7 (87.5%)
Does your cluster have an available up-to-date list of national health research priorities for all institutions within the cluster?	2 (25%)	6 (75%)
Is there a structured approach to identify health research priorities?	*2 (25%)	6 (75%)
Is there a structured tool to identify health research priorities?	*2 (25%)	6 (75%)

*Institution-specific approach and tool

**Table 5:** Ethics review structures

	*N* (%)
Is there an ethical review board to evaluate the ethical conduct of health research?	8 (100%)
Is the ethical review board centralized across the cluster or institution-specific?	
Centralized	4 (50%)
Institution-specific	3 (37.5%)
Not applicable	1 (12.5%)
Is researcher training or certification on ethical research conduct a requirement within your cluster?	8 (100%)
Does your cluster have a unified policy on conflict of interest in research?	5 (62.5%)

### Ethics Review Structures

**Fig. 1:**
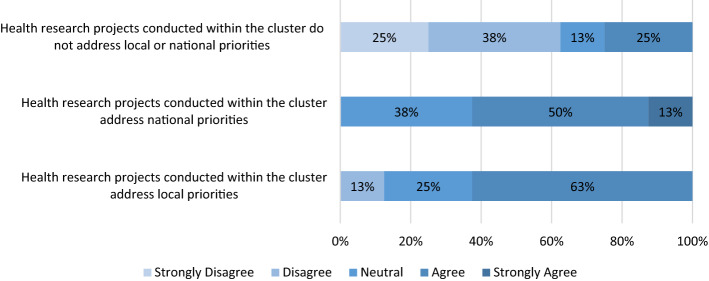
Priority setting for health research in clusters

**Fig. 2:**
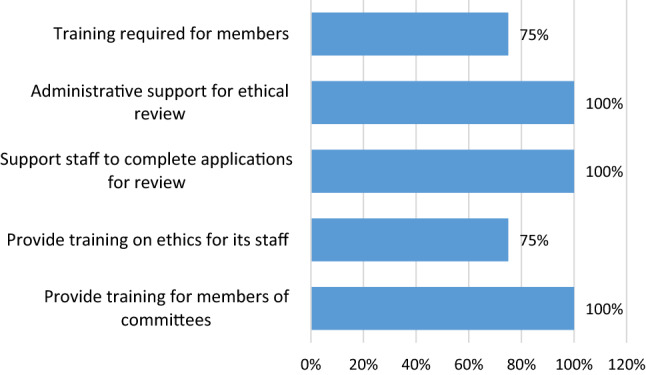
Institutional support for ethical review

All clusters had an ethical review board, which was centralized for 50% and institution-specific for 37.5%. All clusters required researcher training on ethical conduct, and 62.5% had unified conflict-of-interest policies in research. As further detailed in Fig. 1, all clusters train researchers on the ethical conduct of research, assist staff in the review process, and provide administrative support Fig. 2.

### Monitoring and Evaluation

**Fig. 3:**
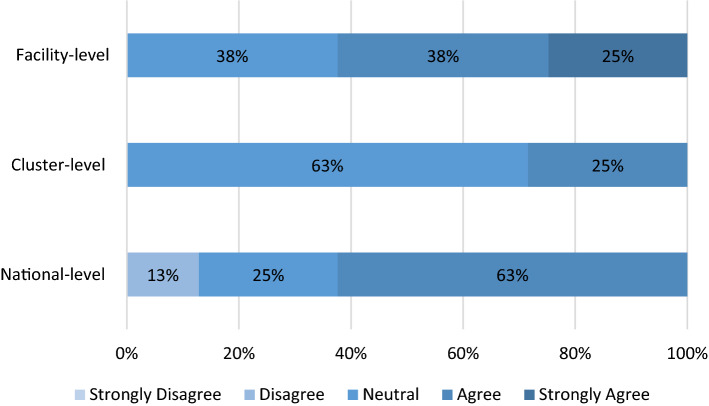
Level of Influence of health research projects on decision-making

Four clusters (50%) reported adopting institution-specific M&E frameworks for the health research system. Regarding key performance indicators (KPIs) for assessing research outputs and outcomes, 25% had cluster-specific KPIs, while 37.5% had institution-specific KPIs. The M&E frameworks covered mostly primary outputs (62.5%) and research processes (50%) (Table 6). In addition, 62.5% of the clusters agreed that health projects had a national impact on decision-making, 62.5% agreed and strongly agreed that the impact was at the facility level, and 25% believed the impact was limited to the cluster level (Fig. 3).

**Table 6:** Monitoring and evaluation

	*N* (%)
*Is there a monitoring and evaluation framework for the health research system?*	
Yes, cluster-specific	2 (25%)
Yes, institution-specific	4 (50%)
No	2 (25%)
*Is there a list of key performance indicators for assessing health research outputs and outcomes?*	
Yes, cluster-specific	2 (25%)
Yes, institution-specific	3 (37.5%)
No	3 (37.5%)
*Which of these dimensions does the monitoring and evaluation framework cover?*	
Research inputs (i.e.,., costs and resources required to conduct project)	2 (25%)
Research processes (i.e.,., efficiency and standards compliance of core research activities)	4 (50%)
Primary outputs (i.e.,., publications, intellectual property filings, conference presentations)	5 (62.5%)
Adoption (i.e.,., number of hospitals or facilities that adopt a specific type of research innovation, number of policies informed by the research)	1 (12.5%)
Research impact (i.e.,., on health and economic outcomes)	–

### Health Research Funding

**Fig. 4:**
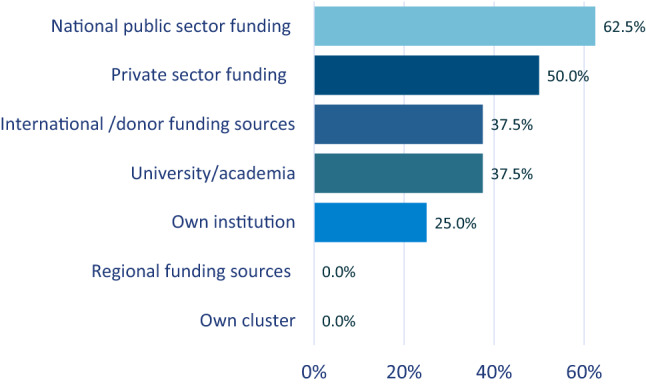
Types of research funding

The most common funding sources were the national public and private sectors (Fig. 4). Few clusters reported being funded by international donors or their own institutions.

Table 7 shows that 42.9% of the clusters collaborated with universities for funding opportunities, and a similar proportion reported institutional collaboration for extramural grants, which only one cluster succeeded in securing. Meanwhile, one cluster reported establishing a clear mechanism for communicating cluster research needs to funders, while another had a similar process for institutional research. Only one cluster had a committed budget line for research (Table 7).

**Table 7:** Health research funding

To what extent do you agree with the following?	Strongly disagree	Disagree	Neutral	Agree	Strongly agree
	*N* (%)	*N* (%)	*N* (%)	*N* (%)	*N* (%)
The institutions within my cluster have a committed budget line for health research	2 (28.6%)	3 (42.9%)	1 (14.3%)	1 (14.3%)	–
There is a clear mechanism to communicate *institutional* health research needs to funders	1 (14.3%)	4 (57.1%)	1 (14.3%)	1 (14.3%)	–
There is a clear mechanism to communicate *cluster* health research needs to funders	1 (14.3%)	4 (57.1%)	1 (14.3%)	1 (14.3%)	–
My cluster has been successful in securing adequate extramural research grants	–	4 (57.1%)	2 (28.6%)	1 (14.3%)	–
Institutions within my cluster collaborate together to apply for joint extramural research grants	1 (14.3%)	2 (28.6%)	3 (42.9%)	1 (14.3%)	–
Institutions within my cluster collaborate with other universities and external partners to apply for joint funding opportunities	–	2 (28.6%)	2 (28.6%)	3 (42.9%)	–

### Resources, Training, and Capacity Building

**Fig. 5:**
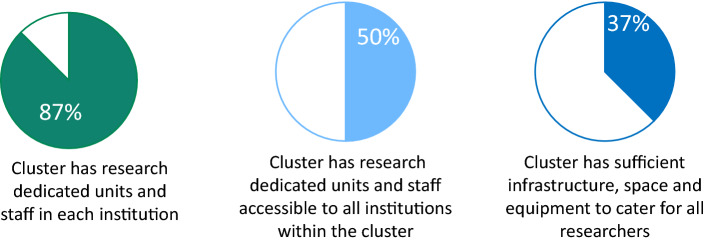
Resource availability

Among the research clusters, the majority have dedicated research units, and 50% have units and staff accessible to all cluster institutions (Fig. 5). Three of the eight clusters (37.5%) reported offering sufficient infrastructure, space, and equipment to cater to all researchers. More than two-thirds of the clusters stated that they have established assessment procedures for research capacity: two at the cluster level and three at the institutional level. Half of the clusters offered formal continuing education and training at the institutional level, and a similar proportion evaluated needs and capacity at the institutional level (Table 8).

**Table 8:** Resources, training, and capacity building

	*N* (%)
Are there established procedures for assessing the research capacity needs of both new and long-term staff?	
Yes, cluster-specific	2 (25%)
Yes, institution-specific	3 (37.5%)
No	3 (37.5%)
Do you offer formal continuing education and training programs on research?	
Yes, cluster-specific	1 (12.5%)
Yes, institution-specific	1 (12.5%)
Yes, cluster- and institution-specific	4 (50%)
No	2 (25%)
Do you assess staff needs and capacity before engaging in capacity building and training?	
Yes, cluster-specific	1 (12.5%)
Yes, institution-specific	1 (12.5%)
Yes, cluster- and institution-specific	4 (50%)
No	2 (25%)
Does your cluster develop research careers for all key health professions?	
Yes	1 (12.5%)
No	3 (37.5%)
Unsure	4 (50%)
What incentives are available to encourage clinicians and staff within your cluster to engage in health research?	
Staff members are provided with protected time to conduct health research	3 (37.5%)
Staff members are rewarded financially for engaging in health research	1 (12.5%)
Conducting health research is considered as part of the performance appraisal of staff	3 (37.5%)
Authorship and publication opportunities	6 (75%)
Awards and recognition	4 (50%)
Based on your clinician and staff profile in your cluster, which of the following areas should be prioritized in training programs?	
Identifying priority topics for health research	7 (87.5%)
Conducting health research (including analysis)	6 (75%)
Developing proposals	6 (75%)
Publishing research	6 (75%)
Translating research findings into practice and policy	6 (75%)
Engaging policymakers and stakeholders in health research	6 (75%)
Monitoring and evaluation of health research outputs and impact	7 (87.5%)

**Fig. 6:**
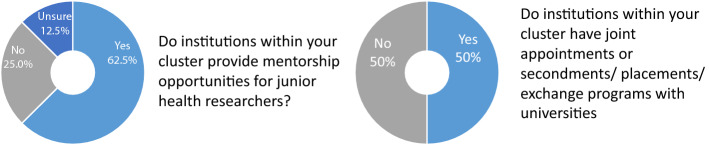
Support for researchers

One cluster reported developing research careers for all key professions. Meanwhile, 62.5% have established mentorship opportunities for junior researchers, and 50% engaged in collaborations with academic institutions for appointments or secondments/placements of staff. Some main incentives were awards and recognition, authorship, and publication; less frequently used ones included research as part of performance appraisal, financial rewards, and protected research time (Fig. 6).

### Health Research Production and Use

**Fig. 7:**
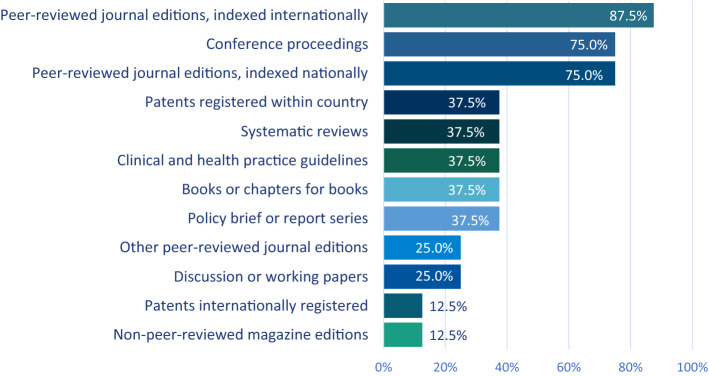
Information products generated by clusters

Most clusters disseminate research findings via peer-reviewed journals (national and international) and conference proceedings (Fig. 7). Three clusters followed a structured process in sharing research with institutions within the cluster or entities beyond it, and a similar proportion implemented an organized system for sharing outputs with policymakers and stakeholders (Table 9).

**Table 9:** Research production and use

	*N* (%)
Is there a structured process for sharing health research outputs with different institutions within the cluster?	
Yes	2 (25%)
No	6 (75%)
Is there a structured process for sharing health research outputs with key policymakers and stakeholders?	
Yes	3 (37.5%)
No	5 (62.5%)
Is there a structured process for sharing health research outputs with other entities beyond the cluster?	
Yes	1 (12.5%)
No	7 (87.5%)

### Open-ended Question

The survey’s open-ended component grouped barriers and facilitators under four main sections: health research stewardship and governance, health research funding, resources (human and physical), and health research production and use.

Table 10 below shows that the main barriers to health research stewardship and governance were the lack of cluster-level overarching strategies and coordinating bodies. At the institutional level, clusters mostly discussed the lack of resources, policies, and research commitment. These barriers were addressed by establishing cluster-wide research programs with unified policies, conducting cluster-wide priority settings, and M&E.

**Table 10:** Key findings from the open-ended component

Barriers	Facilitators
Health research stewardship and governance	
*At the cluster level* No cluster-specific strategy and infrastructure to conduct research No central oversight body No priority-setting exercises *At the institutional level* Insufficient human resources with required qualifications Lack of institutional policies, guidelines, and procedures Poor research commitment Lack of dedicated units to conduct, follow up, and monitor research activities	Establish a cluster research program with unified policies, procedures, and guidelines with clear governance structures and standard operating procedures (SOPs) Identify a model institution that clusters can refer to Expand the scope of human research protection program (HRPP) Conduct priority setting at the cluster level Create unified policies for the dissemination of research findings Conduct M&E to ensure that SOPs are applied Hire experienced and qualified staff
Health research funding	
No funding/allocated budget for cluster-level research No fund management mechanism (such as dedicated bank accounts) No secure extramural funds No incentive policies for clinical trial stakeholders and institutional review board (IRB) members Lack of academic collaborations Research department support via annual grants and rewards to encourage researchers to conduct more studies	Create a funding mechanism and dedicated research funds within clusters Dedicate research funds for relevant staff Secure extramural funding and grants Develop and implement a robust auditing process Implement policies and secure funds for obtaining and maintaining lab equipment needed for research Establish policies and dedicated funds for clinical trials and assess the impact of such initiatives at the national level Provide staff incentives to participate in research Establish formal partnerships with academia across regions for research and collaboration opportunities
Resources (human and physical)	
Difficulty in recruiting experienced and qualified staff Lack of qualified staff at institutions Competing priorities for staff members Limited research resources (access to electronic databases, statistical software, etc.) Lack of research facilities	Provide staff time and resources to conduct research (including protected research time for physicians) Establish research facilities Attract, recruit, and retain qualified staff from within and outside the region Develop staffing plan for research Establish research mentorship program and provide capacity-building activities Develop certification program for research Develop active internal and external communication strategy Establish national and international research collaborations Institutionalize a research culture
Health research production and use	
Studies conducted are at a small scale Lack of policies to support research efforts Lack of formal systems for disseminating research findings at the cluster level Research not conducted according to regional priorities Lack of translational research strategy Need for better training, support, and funding for clinical trials	Establish research dissemination channels Develop research products such as policy briefs to ensure the uptake of research findings Establish a national research journal Identify research priorities and share them with institutions Develop an M&E program with clear KPIs for research performance Perform a strength, weaknesses, opportunities, and threats (SWOT) analysis of health research Standardize data management Establish a research advisory board Promote multicenter collaborations for large-scale research projects

In terms of health research funding, the main barriers according to the respondents were the lack of funding, grant management mechanisms, incentive policies, and academic collaborations. Facilitators to counteract these barriers included the development of funding mechanisms, dedicating research funds for staffing, developing and implementing policies to support research funding, providing staff incentives, and establishing partnerships with academia.

With regard to resources, the main challenges included difficulties in identifying qualified staff, competing priorities of existing staff, limited resources, and lack of research facilities. Some facilitators to address these issues included recruiting qualified staff, allocating time and incentives for staff to conduct research, establishing research facilities, developing staffing plans, and developing mentorship opportunities.

For health research production and use, reported obstacles included the lack of policies to support research, formal dissemination mechanisms, and translational strategy and the misalignment of research and priorities. To address these issues, measures included establishing formal dissemination channels, developing knowledge translation products, conducting cluster-wide priority-setting exercises, and promoting multicenter collaborations.

## Discussion

This study summarizes the first efforts to assess the capacities, resources, and needs of KSA’s research clusters at baseline. The findings can fill a significant knowledge gap on the requirements for establishing good research governance structures and evaluating the available groundwork to ensure their success. Research governance must be implemented at all stages of a study and apply research principles, standards, and requirements, as well as promote good research culture and practice [14].

As discussed in the results section, in terms of resources, capacity, research portfolio, and policies, some clusters are more advanced than others. There is much that these clusters can do to help the newer clusters succeed in initiating their research agendas, expanding their research portfolios, and developing effective policies. One of the major hindrances preventing clusters from engaging in broader areas of research relates to funding. The majority of clusters obtained funding from local sources which may be limited and focused on specific issues. This may explain the reason why there is limited translational research and a greater focus on basic clinical research. Greater efforts should be made to support researchers in applying for international funding opportunities which focus on broader research topics, reflect priority research and policy areas, and engage multiple clusters. This can build upon existing capacities and fill important national evidence gaps.

There is room to capitalize on the identified strengths in the capacity of health clusters. For instance, most clusters had policies in place on research ethics, and while some were at the cluster level, many were at the institutional level. There is room to expand and adapt these policies as well as share knowledge in this regard, particularly in areas where some clusters lack capacity or knowledge whereas others have more expertise. Most clusters had dedicated research unit in addition to space and infrastructure which can form an important focal point that can support other clusters. There is a rich and diverse cadre of staff that can support cluster research agendas. Clusters can capitalize on such resources locally and establish processes for exchange with universities and research institutions in other clusters. Clusters can also further invest in providing protected time for research and incorporating it in annual appraisals. Financial rewards and recognition can also be used as incentives to encourage researchers to disseminate research findings and support policy and decision making at the national level. Research evidence attests to the fact that providing researchers with such incentives can improve their productivity and support them in attracting additional funding [10].

The results presented a need to develop unified health research guidelines, as several clusters lack some crucial procedures on quality assurance and improvement; bio-specimen access, use, and retention; risk management, privacy, and safety; clinical trials; research misconduct; sponsorship for health research; and research dissemination. Developing national guidelines can support the “newer” clusters in advancing their research profiles and more effectively organizing and implementing research activities.

For effectively governed research, projects should be performed consistently with recognized ethical principles, guidelines for accountable research behavior, applicable legislation and rules, and institutional policy. This also includes conducting research training and capacity building as well as providing them the necessary credentials to research and oversee institutional risks (Australian Government, 2019). In this context, clusters can work towards centralizing ethical review boards and making sure their guidelines and requirements are effectively communicated and uniformly implemented in all institutions.

With respect to research ethics, all clusters had ethical boards, with most having guidelines in place to support and train staff in this aspect. However, one area that needs work is the conflict-of-interest policy. Issues around conflict of interest are gaining more attention, as they can influence research conduct and the reporting of results. Hence, the reporting of personal, political, industrial, academic, and other conflicts must be declared and managed [4].

Regarding the setting of priorities, clusters reported using tools for conducting such exercises but no entities that would identify and fund such priorities. With the growing number of studies, ensuring the efficient and targeted funding of areas where research is actually needed is crucial. Installing structured prioritization processes and structures can help generate research evidence that would fill significant knowledge gaps and support the health policy and decision-making process [8]. Working directly with policymakers and research users and identifying contextual population needs can support such efforts [8]. In fact, priority setting should incorporate different stakeholders’ values to overcome fundamental challenges to the healthcare system while ensuring the efficient use of finite resources [7].

In terms of M&E, few clusters had indicators for assessing outputs, which can provide much-needed information on accountability, efficiency, resource allocation, and improvement points [13]. Furthermore, information generated from evaluation efforts can be used to promote existing processes and generate evidence more efficiently [13].

## Conclusion and Implications

The present findings clearly show strong efforts to support research governance initiatives in health clusters in KSA. While some clusters are more advanced than others, there are plenty of opportunities to share knowledge and combine efforts to help achieve the goals set out for KSA health transformation. The lessons learned from existing local efforts can be used to advance other clusters’ efforts and allow them to reach their goals faster and with fewer resources. Addressing priority areas and generating research evidence to fill knowledge gaps can significantly improve the policymaking process and strengthen the health system in KSA. Clusters can start working on the findings generated from this study to support existing efforts to strengthen their respective research infrastructures.

The results of this baseline assessment also reflect the first attempt of its kind to understand the KSA experience and provide much-needed lessons on country-wide efforts to support the health system given the trickling effect of this sector on all others, enhancing and advancing national growth. Countries in the region engaging in health reform or planning can benefit from this study’s findings to support assessment efforts, identify available resources, and develop action-oriented plans for health reform.

## Data Availability

Data and material are available on request.

## Abbreviations

KSA: Kingdom of Saudi Arabia
NHMRC: National Health and Medical Research Council
NICE: National Institute for Health and Care Excellence
M&E: Monitoring and evaluation
COVID-19: Coronavirus disease 2019
MERS-Cov: Middle East Respiratory Syndrome coronavirus
IP: Intellectual property
KPIs: Key performance indicators
HRPP: Human research protection program
SOPs: Standard operating procedures
IRB: Institutional review board
SWOT: Strength, weaknesses, opportunities, and threats analysis
